# Effects of Royal Jelly Freshness and Concentration on Lifespan, Growth, Motility and Reproduction in *Caenorhabditis elegans*

**DOI:** 10.3390/foods14223839

**Published:** 2025-11-10

**Authors:** Chenhuan Zhang, Yuanhao Deng, Zhenling Luo, Shenyun Liu, Wenhui Tao, Yuhan Zhang, Hongliang Li, Fan Wu

**Affiliations:** 1 Zhejiang Provincial Key Laboratory of Biometrology and Inspection and Quarantine, College of Life Science, China Jiliang University, Hangzhou 310018, China; 2 Taizhou Institute of Food and Drug Control, Taizhou 318100, China

**Keywords:** royal jelly (RJ), freshness, longevity, reproductive capacity, *Caenorhabditis elegans*

## Abstract

Although aging is an irreversible process, the rate of aging can be delayed by a reasonable diet. As a nutrient-dense natural product, royal jelly (RJ) has an enormous potential for applications in medicine and health promotion. However, the exact physiological activity of RJ with varying freshness and concentration has not been fully clarified, and more investigation is needed to determine their precise contributions. Here, fresh RJ (just produced recently) and RJ stored for 2 weeks at −20 °C, 4 °C or 25 °C were tested at concentrations of 100, 50, 25 and 12.5 μg/mL on *Caenorhabditis elegans*. Fresh RJ, with concentrations of 100 μg/mL, 50 μg/mL and 25 μg/mL, could extend the lifespan of *C. elegans* by 16.37%, 9.53% and 4.32%, while RJs stored at 4 °C and 25 °C were ineffective. In terms of body length, treatment with fresh RJ significantly enlarged the body size by around 48%. Although RJ stored at 4 °C and 25 °C could also promote nematode growth, its activity diminishes as storage temperature increases. RJs stored at −20 °C and 4 °C with concentrations of 100 μg/mL significantly increased the pumping rate of nematodes by 58% and 50%. But non-fresh RJ or low-concentration RJ (≤25 μg/mL) had no effects on the motility of *C. elegans*. In addition, fresh RJ could improve the reproductive capacity of *C. elegans*, with the highest increase reaching approximately 25%. Even when stored at 25 °C, RJ also significantly enhanced the reproductive capacity of *C. elegans*, increasing it by approximately 14.8%. Moreover, qPCR showed that RJ could significantly affect the expression of multiple genes associated with aging and vitality. Fresh RJ significantly up-regulated *bec1* and *hsp16.2* 3.19- and 2.80-fold, while RJ stored at 25 °C significantly up-regulated *sod3* and *gpd1* 3.80- and 3.40-fold. Our results suggested that the activity of RJ on *C. elegans* is related to its freshness and concentration, while RJ also contains active components that are independent of freshness. Therefore, it is necessary to explore effective methods for accurately assessing the freshness of RJ.

## 1. Introduction

The process of physiological function decline is known as aging, and it has garnered attention throughout human history [1]. Although aging is a natural occurrence, a number of factors, including stress and unhealthy diet and living habits, are linked to accelerating aging by exacerbating chronic subclinical inflammation [2,3]. Certainly, it is possible to delay the onset of aging and related diseases through the consumption of nutriments and bioactive substances [4,5].

Royal jelly (RJ), a white jelly-like substance, is secreted from the hypopharyngeal and mandibular glands of nurse bees (*Apis mellifera ligustica*) [6]. RJ is consumed by the queen and larvae within 3 days, and plays a key role in the caste differentiation of honeybees [7,8]. In addition, RJ can also enhance the reproductive ability of the queen. Due to its biological activity, RJ has also been used in traditional medicine. In many nations, particularly in Asia and Europe, it is frequently advertised and sold as a “health” food and cosmetic [9,10]. A comprehensive study of the biology, chemistry, pharmacology and clinical trials of RJ has been available for a few decades, and many physiological and functional activities have also been published [11,12]. These activities include vasodilative, hypotensive, anti-allergenic, anti-fatigue, anti-inflammatory and anti-tumor properties [13,14,15]. RJ is made up of a variety of essential compounds, including proteins, lipids, vitamins, free amino acids, flavonoids and polyphenols [16]. And the composition of RJ varies with season and the resources of plants around the apiary [17]. Protein accounts for about 50% of the dry weight of RJ; more than 80% of RJ proteins are major royal jelly proteins (MRJPs; MRJP1-9 have been reported) [18]. Research has shown that the broad spectrum of physiological activity of RJ is associated with protein components [19]. Due to the instability of proteins and macromolecules, RJ is highly degradable and perishable [20]. And degraded RJ loses much of its biological functions. Therefore, RJ may spoil and lose these health-promoting properties depending on storage duration and conditions [21].

The properties of *C. elegans* make it useful for studying a wide variety of diseases and biological processes, especially in aging research [22]. In order to investigate the dynamic activity changes in storage temperature and concentration, RJs stored at −20 °C, 4 °C and room temperature (25 °C) for several weeks were used to treat *C. elegans*. Then, we statistically analyzed the lifespan, growth, motility and reproductive capacity of L4 stage worms. Finally, the gene expression levels in pathways related to antioxidants, homeostasis maintenance and lipid metabolism were detected using fluorescence quantitative PCR. This study will provide the theoretical basis for fully exploiting the interaction of the freshness of RJ and its bioactivity.

## 2. Material and Methods

### 2.1. Materials

The wild-type *C. elegans* strains (wild-type, Bristol N2) and *Escherichia coli* OP50 (*E. coli* OP50) were maintained in our laboratory [23]. The eggs were incubated on nematode growth medium (NGM) agar plates at 20 °C and seeded with a live *E. coli* OP50 as a food resource [23]. CaCl_2_, MgSO_4_, peptone, NaCl, agar, PBS buffer, tryptone, yeast extract, NaOH, KH_2_PO_4_, NaClO and anhydrous ethanol were purchased from Sangon Biotech Co., Ltd. (Shanghai, China). The RNA extraction kit and the reverse transcription kit were purchased from Takara biotechnology Co., Ltd. (Dalian, China). All reagents used were of analytical grade. NaOH and household bleach were applied for bleaching the worms.

### 2.2. Preparation of RJ and Treatment Plates

The experimental colonies of honeybees were maintained at China Jiliang University (Hangzhou, China). Larvae less than 24 h old were transferred into plastic queen cups and returned to the colonies. Fresh RJ was collected approximately 68–72 h post-grafting. The RJ samples were promptly transferred into 50 mL Eppendorf tubes and subsequently stored at temperatures of −20 °C, 4 °C and 25 °C for two weeks. Before the experiment, the royal jelly samples were rapidly thawed at room temperature, and 1 g was weighed using a one-thousandth precision balance. Then, the royal jelly samples were diluted with PBS as needed.

### 2.3. C. elegans Maintenance, Synchronization and Feeding RJ

*C. elegans* were maintained on nematode growth medium (NGM) plates, which were seeded with *Escherichia coli* OP50 and cultured at 20 °C. To obtain synchronized *C. elegans*, gravid adult nematodes were lysed using a lysis buffer consisting of 20 mg/mL NaOH, 1 mL of 5% NaClO and 9 mL of ddH_2_O and then hatched overnight on sterile NGM. The released eggs were then washed twice with M9 buffer (composed of 6 g/L Na_2_HPO_4_, 3 g/L KH_2_PO_4_, 5 g NaCl and 0.25 g/L MgSO_4_·7H_2_O). These eggs were subsequently cultivated on NGM plates until they reached L1 larval stage. L1 stage worms were then randomly transferred to fresh NGM plates. The control group was treated with PBS, while experimental groups were administered RJ of varying freshness and concentrations (100 μg/mL, 50 μg/mL, 25 μg/mL, 12.5 μg/mL). Unless otherwise stated, all other experimental conditions were exactly the same. The treatment was administered continuously for three days. In the experiments assessing life span, growing ability, motor ability and reproduction, treatments started from hatching unless otherwise stated.

### 2.4. Lifespan Assays

For each experiment, we placed 15 L4-stage larvae on three plates with 5 animals per plate and allowed these to develop to adulthood and then lay eggs over 24 h. These parental animals were then removed from the plates. After 48 h, 60 L4 larvae were transferred to fresh plates. These animals were transferred to fresh plates every day during the progeny production period, and then every other day thereafter. Animals were assessed as dead if they remained motionless when gently poked. Lifespan numbers did not include animals that moved off the plate or perished from internal hatching or vulva bursting.

### 2.5. Growing Ability Assay

Synchronized worms were cultured with RJ at various storage temperatures and dilution ratios until adult stage (L4), after which they were transferred to a blank NGM for 1 min acclimation period. The worms were then anesthetized with ethanol for 1–2 min to allow full body extension, after which their body length was measured using a Nikon SMZ745T with a calibrated scale.

### 2.6. Motor Ability Assay

The synchronous nematodes of L4-stage larvae were transferred to NGM plates with different concentrations of RJ and were transferred every 24 h to the fresh plates. The sinusoidal movement was observed for 30 s with a camera equipped with an anatomical microscope and streamplx imaging. The movement of the head was observed as it oscillated from one side of the body to the other and returned to its original position. All assays were carried out in triplicate.

### 2.7. Fecundity Assay

Animals were raised from eggs, similarly to in the lifespan assays. Synchronized worms were cultured until adult stage (L4), after which they were transferred to a blank NGM. Worms were transferred to blank NGM plates daily until egg-laying ceased. The number of viable eggs to hatch each day was recorded. Data shown represent the average of three independent trials.

### 2.8. RNA Extraction and Quantitative Real-Time PCR (RT-qPCR)

Worms from each treatment group were collected and washed with M9 buffer. The samples were then homogenized, and total RNA was extracted using an RNA extraction kit. RNA concentrations were measured with NanoDrop 2000 (Thermo Scientific, Waltham, Massachusetts, USA). Reverse transcription of the total RNA was performed using a reverse transcription kit. Quantitative PCR was conducted using the TB Green™ Premix Ex Taq™ II (Tli RNaseH Plus) kit, with the primer sequences listed in Table 1 and β-actin as the reference gene. The expression levels of genes related to antioxidation, homeostasis and lipid metabolism in *C. elegans* were calculated using the 2^−^^ΔΔCt^ method, with the PBS-treated group serving as the baseline.

### 2.9. Statistical Analysis

All experimental data are shown in the text as mean ± standard deviation (SD, *n* = 3). Statistical analyses between the control and treated group were performed using one-way analysis of variance (ANOVA) in SPSS 27.0 software (SPSS Inc., Chicago, IL, USA), with significance determined at *p* < 0.05.

## 3. Result

### 3.1. RJ Slightly Increased the Lifespan of C. elegans

**Figure 1:**
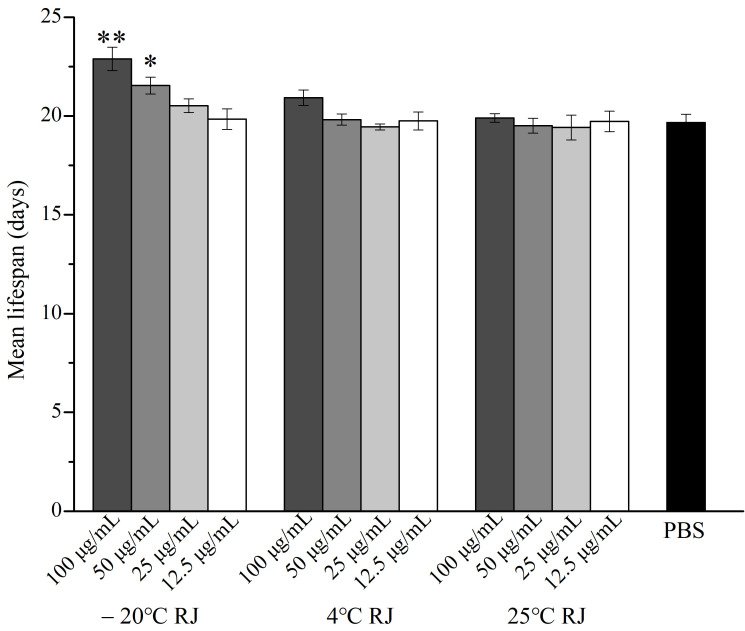
Effect of RJ at different storage temperatures and dilution ratios on the lifespan of *C. elegans*. Data were expressed as the mean ± SD. Bars with different asterisks indicate statistical significance (* *p* < 0.05, ** *p* < 0.01).

Under the experimental conditions, the effect of different storage temperatures and dilution ratios of RJ on the lifespan of *C. elegans* are shown in Figure 1 and Table 2. During the experiment, the data of the nematodes that died due to operational errors or accidents were excluded. After administering with 100 μg/mL, 50 μg/mL and 25 μg/mL RJ stored at −20 °C, the mean lifespan of *C. elegans* increased to 22.89 ± 0.59, 20.68 ± 0.73 and 20.52 ± 0.34 days, respectively, which expanded the lifespan of *C. elegans* by 16.37%, 9.53% and 4.32%, respectively, compared to the control group. However, RJ stored at 4 °C and 25 °C did not affect the lifespan of *C. elegans*. These results suggested that only fresh RJ can prolong the lifespan of *C. elegans*.

### 3.2. Effects of Different RJ on Body Length of C. elegans

**Figure 2:**
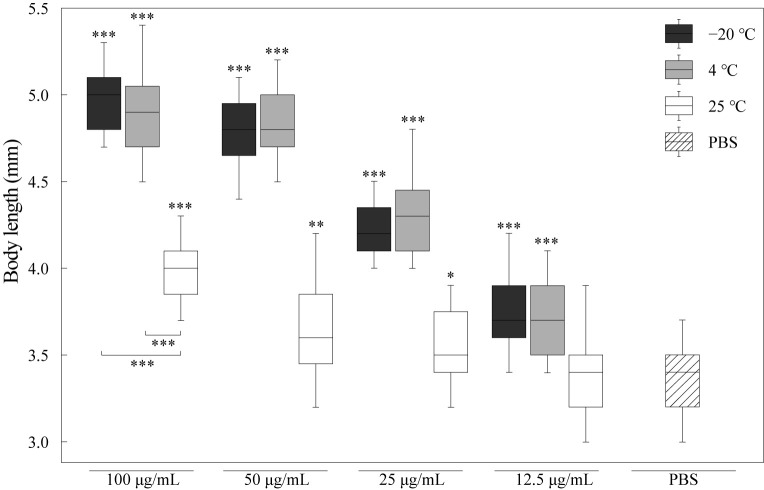
Effect of RJ at different storage temperatures and dilution ratios on the body size of *C. elegans*. Data were expressed as the mean ± SD (*n* = 3). Bars with different asterisks indicate statistical significance (* *p* < 0.05, ** *p* < 0.01 and *** *p* < 0.001).

We determined and analyzed the effects of RJ on the body length of nematodes, as shown in Figure 2. Compared with the control group, the body size of the nematodes in the RJ treatment groups were significantly bigger. And the body size increased by 48%, 42%, 26% and 13% after being treated with 100 μg/mL, 50 μg/mL, 25 μg/mL and 12.5 μg/mL RJ stored at −20 °C. Certainly, the RJ samples stored at 4 °C and 25 °C could also promote nematode growth, but the activity decreased with the increasing storage temperature. These results indicated that the component of RJ could improve the nematode growth rate, and the nutrients that provide energy have relatively low requirements for storage temperature. However, the nutritional components will still degrade when stored at 25 °C.

### 3.3. RJ Improved the Motility of C. elegans

**Figure 3:**
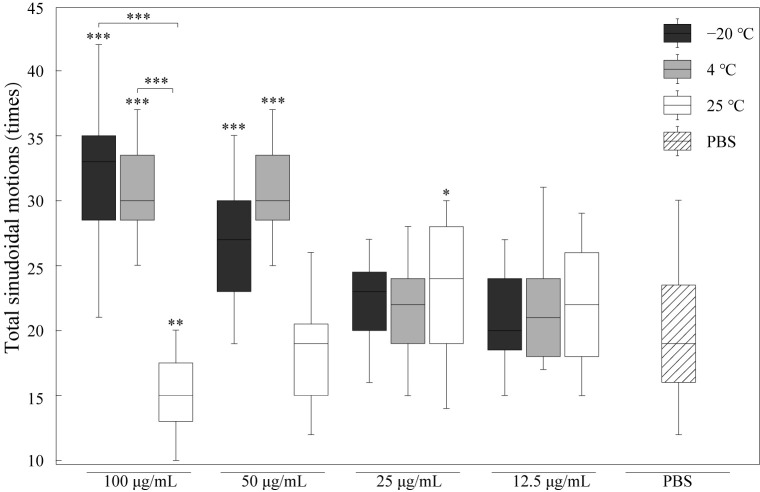
Effects of RJ at different storage temperatures and dilution ratios on motility of *C. elegans*. Data were expressed as the mean ± SD (*n* = 3). Bars with different asterisks indicate statistical significance (* *p* < 0.05, ** *p* < 0.01 and *** *p* < 0.001).

Locomotion behavior is commonly used as a marker of aging. To analyze the effect of RJ on the locomotion behavior of nematodes, we tested the motility of nematodes treated with RJ stored at −20 °C, 4 °C and 25 °C, as shown in Figure 3. RJ stored at a low temperature (−20 °C and 4 °C) with a concentration of 100 μg/mL significantly increased the locomotion behavior of nematodes by 58% and 50% compared to the control group. However, when the concentration of royal jelly is lower than 25 μg/mL, its promoting effect on the motility of *C. elegans* is not significant. In addition, RJ stored at 25 °C for two weeks lost its biological activity in promoting nematode motility. These results suggested that only fresh high-concentration RJ could improve the motility of worms.

### 3.4. RJ Showed Significant Effect on the Reproduction Capacity of C. elegans

**Figure 4:**
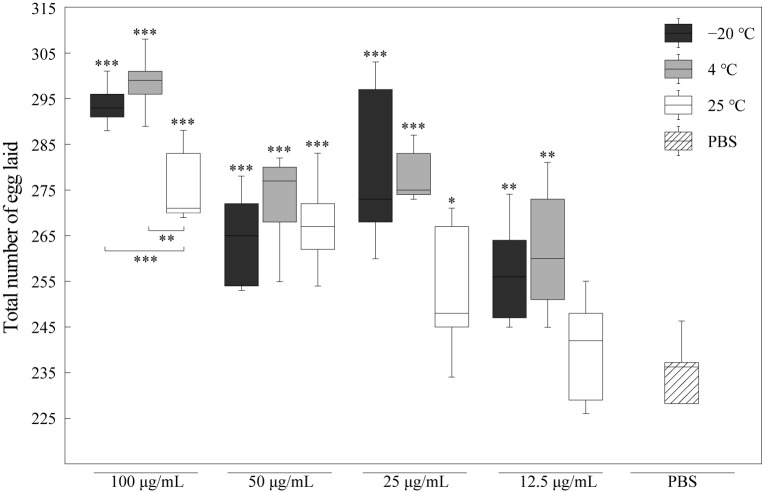
Effects of RJ at different storage temperatures and dilution ratios on fecundity of *C. elegans*. Data were expressed as the mean ± SD (*n* = 3). Bars with different asterisks indicate statistical significance (* *p* < 0.05, ** *p* < 0.01 and *** *p* < 0.001).

According to the trade-off between reproduction and longevity theory, the lifespan is enhanced if resources shift from somatic maintenance to reproduction. To evaluate whether the difference in lifespan induced by RJ was accompanied by an over-promotion of individual vitality, we further analyzed the change in the reproduction capacity of nematodes treated with RJ stored at −20 °C, 4 °C and 25 °C, as shown in Figure 4. Compared to its effects on lifespan, RJ has a more significant effect in promoting the reproductive capacity of *C. elegans*. The effects of different concentrations of RJ stored at −20 °C and 4 °C on the reproductive capacity of *C. elegans* were significantly higher than those of the PBS control group, with the highest increase reaching approximately 25%. Even when stored at 25 °C, 100 μg/mL RJ also significantly enhanced the reproductive capacity of *C. elegans*, increasing it by approximately 14.8%.

### 3.5. Aging-Related Gene Expression

**Figure 5:**
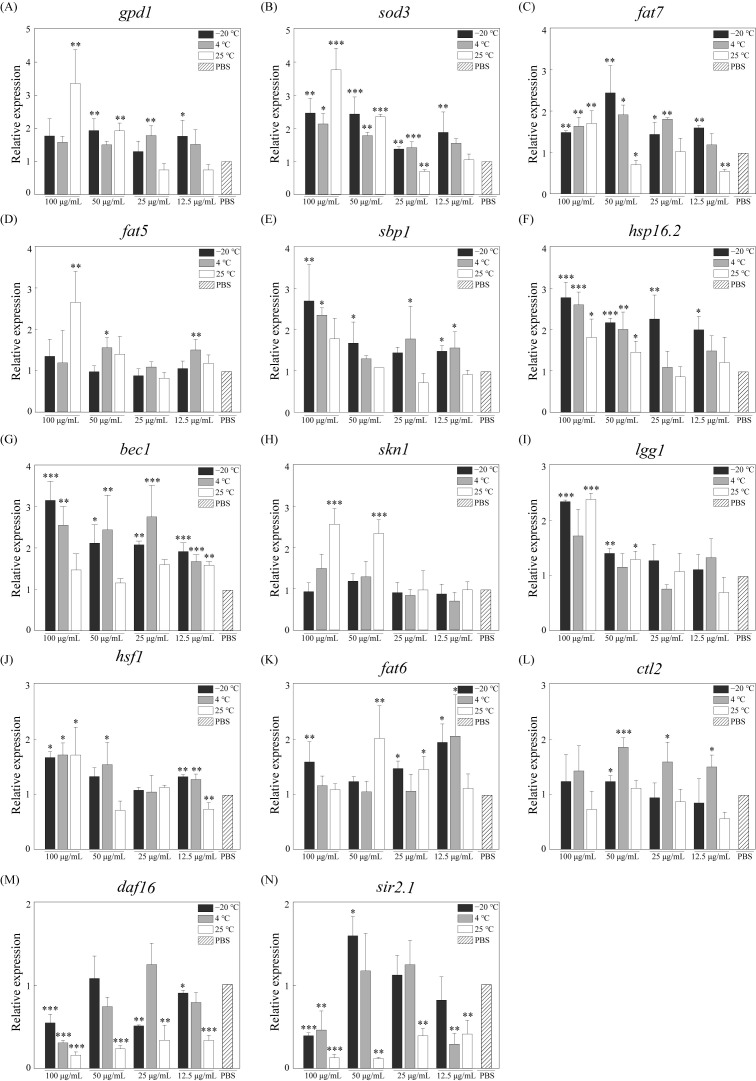
Relative expression levels of aging- and vitality-related genes in *C. elegans* after treatment with RJ at various storage temperatures and dilution ratios. (**A**) *gpd1*, (**B**) *sod3*, (**C**) *fat7*, (**D**) *fat5*, (**E**) *sbp1*, (**F**) *hsp16.2*, (**G**) *bec1*, (**H**) *skn1*, (**I**) *lgg1*, (**J**) *hsf1*, (**K**) *fat6*, (**L**) *ctl2*, (**M**) *daf16*, (**N**) . * *p* < 0.05, ** *p* < 0.01 and *** *p* < 0.001.

The above-mentioned results showed that RJ could improve the physiological function of *C. elegans*, including accelerating growth, raising the motility, increasing the fecundity and prolonging the lifespan, but the mechanisms are still not clear. Therefore, we analyzed the expression levels of genes associated with aging and vitality, as shown in Figure 5. The gene expression of the control group was defined as 1. The experimental results showed that RJ could significantly affect the expression of multiple genes associated with aging and vitality. RJ stored at −20 °C and RJ stored at 4 °C exhibit similar trends in regulating the genes associated with *C. elegans*. In the experimental group treatment with RJ stored at −20 °C, the relative expression levels of *sod3*, *lgg1*, *bec1*, *hsf1*, *hsp16.2*, *sbp1* and *fat7* were significantly up-regulated 2.49-, 2.36-, 3.19-, 1.69-, 2.80-, 2.72- and 1.52-fold, while the relative expression levels of *sir2.1* and *daf16* were down-regulated 0.41- and 0.57-fold. In the experimental group treatment with RJ stored at 4 °C, the relative expression levels of *sod3*, *lgg1*, *bec1*, *hsf1*, *hsp16.2*, *sbp1* and *fat7* were significantly up-regulated 2.17-, 1.74-, 2.59-, 1.73-, 2.63-, 2.38- and 1.67-fold, while the relative expression levels of *sir2.1* and *daf16* were down-regulated 0.48- and 0.32-fold. In the experimental group treatment with RJ stored at 25 °C, the relative expression levels of *skn1*, *sod3*, *lgg1*, *hsf1*, *hsp16.2*, *fat7*, *fat5* and *gpd1* were significantly up-regulated 2.58-, 3.80-, 2.40-, 1.73-, 1.84-, 1.73-, 2.68- and 3.40-fold, especially genes related to lipid metabolism, while *sir2.1* was significantly down-regulated 0.14-fold. In addition, different dilution ratios also lead to differences in the regulation of gene expression level in *C. elegans*. Overall, high concentrations of RJ have a stronger effect on *C. elegans*.

## 4. Discussion

Though aging or hypokinesis is a natural phenomenon, numerous factors such as stress, poor nutrition and pollution are associated with the increased internal production of free radicals, which enhance chronic subclinical inflammation and lead to faster aging. Therefore, one of the biggest challenges in life sciences is figuring out how to postpone the beginning of aging and related disorders. RJ is one of the most important products of bees, and is used to feed the queen and larvae within 3 days. And it has also been used as a natural health supplement by the elderly for several decades. Reports have revealed that RJ exhibits several physiological activities in experimental animals, including vasodilative and hypotensive activities, increases in growth rate, disinfectant actions, antitumor activity and anti-inflammatory activity. Although it is widely acknowledged that the activity of RJ is closely linked to its freshness, research in this area remains insufficient. To investigate the dynamic activity changes in storage temperature, the RJ stored at −20 °C, 4 °C and 25 °C were used to treat *C. elegans*. The results suggest that the activity of RJ on *C. elegans* is related to its freshness, while RJ also contains active components that are independent of freshness.

Fresh RJ has a promoting effect on enhancing the activity of *C. elegans*. In a honeybee colony, although the queen and the worker bees share the same genome, the queen enjoys a significantly longer lifespan due to consuming RJ throughout its entire life. And RJ was also reported to contribute to the prolongation of longevity in other animals [24,25]. It has been reported that intermediate and high doses (50 and 500 ppm) of RJ increased the average lifespan of mice, possibly through the mechanism of reduced oxidative damage [26]. Researchers also found that protease-treated RJ and royalactin (formerly named 57-kDa protein) extended the lifespan of *C. elegan* [27]. In our study, compared to the control, fresh RJ at 100 μg/mL and 12.5 μg/mL can significantly prolong the lifespan of *C. elegans*. This suggests that RJ spoils and loses the properties to extend lifespans depending on storage conditions. Nutritionally, the chemical composition of RJ is one of the most complex, with proteins accounting for approximately 50% of its dry weight; about 80–90% of RJ proteins are MRJPs, including MRJP1-9 [18]. Protein components play a key role in the biological activity of RJ. However, RJ proteins are extremely sensitive to storage temperature. For example, MRJP1 is degraded proportionally to the timescale of storage and is completely lost after storage for 30 days at 40 °C [8]. MRJP3, MRJP4 and MRJP5 showed a gradual and remarkable degradation under non-low-temperature storage, and MRJP4 was almost completely degraded after storage for 3 days at 37 °C [21]. Similarly, royalactin is degraded in proportion to both storage temperature and storage period [27]. These studies all indicate that proteins maybe play a significant role in extending lifespan. However, it is challenging to adequately explain the increased longevity depending on a single molecule or mechanism, since it is far more complicated than we realize. We have conducted a preliminary analysis of the changes in proteins and peptides in fresh royal jelly and stored royal jelly. The results showed that the levels of MRJPs, glucose oxidase and apolipoprotein III in royal jelly stored under different conditions had undergone significant changes. But I still do not know the degradation efficiency of these proteins.

Except for components that are sensitive to storage temperature, RJ also contains stable active substances that do not depend on the storage conditions. In most cases, the quality of life is more important than only longevity extension. Another well-known benefit of RJ is its protective effect on reproductive health [11]. Previous studies on male rabbits have demonstrated that RJ exerts positive effects on fertility, semen quality and yield, as well as blood levels of testosterone and total proteins [28]. Recent studies have reported that RJ protects against oxidative injuries in the mouse testes, and that it contains spermatogenesis-stimulating compounds which inhibit the production of proinflammatory cytokines [29]. It is also efficient in preventing the depletion of follicle pool and in enhancing hormonal regulation [11]. In aged rats, RJ can increase the amounts of ovulated oocytes and improve their quality by regulating hormone levels [30]. According to our results, we found that RJ has a more substantial impact on enhancing the reproductive capacity of *C. elegans* than it does on the lifespan. Even when stored at 25 °C, RJ significantly enhances the reproductive capacity of *C. elegans*. This suggested that RJ contains stable active substances that do not depend on the storage conditions. For example, the inverse isomer of (E)-10-hydroxyl-2-decylenic acid (10-HDA) is a unique fatty acid found only in RJ, and is an effective composition used to judge the quality of RJ as well [31]. Through the study using HPLC-MS/MS, it is determined that the content of 10-HDA would not change even after being stored at room temperature for six months [21]. 10-HDA in particular may improve the hormonal balance by enhancing the production of the ovulation hormones and by preserving the follicular pool [32]. Researchers have confirmed the effect of 10-HDA on the reproductive potential in sheep. Apart from 10-HDA, the stable adenosine monophosphate (AMP) N1 oxide, adenosine, acetylcholine, polyphenols and hormones are all useful bioactive components [11]. RJ can not only extend the lifespan of *C. elegans* but can also enhance its reproductive capacity. This situation conforms to the trade-off between reproduction and longevity theory, namely that the lifespan is enhanced if resources divert from somatic maintenance to reproduction [33].

The exact mechanisms of the action of RJ on *C. elegans* have not been fully elucidated, but multiple genes such as *bec1*, *hsp16.2*, *sod3* and *gpd1* are involved. Previous studies have shown that numerous pathways in *C. elegans*, such as the insulin/IGF-1 signaling (IIS) pathway, TOR signaling pathway, gonad signaling pathway, lipid metabolism pathway and mitochondrial electron transport chain, are proved to regulate lifespan and reproductive capacity [34,35,36]. And several best-characterized genes, including *daf16* and *age1*, are involved in the process of lifespan regulation [37,38,39]. Here, we found that fresh and stale royal jelly can regulate the expression levels of different genes in *C. elegans*. This suggested that different components in royal jelly could affect the lifespan, body length, motility and reproduction capacity of nematodes by regulating the expression of antioxidant-, homeostasis- and lipid metabolism-related genes. For example, the *bec1* gene is crucial to *C. elegans* autophagy, which is closely related to mediating lifespan extension [40,41]. The *hsp16.2* gene can mediate the heat-shock signaling pathway, thereby extending the lifespan and enhancing the reproductive capacity of *C. elegans* [42]. Thus, RJ helps in preventing the aging process and is an influential antiaging product; the mechanism of action needs to be further clarified. It is worth noting that the current international trade volume of lyophilized royal jelly is close to that of fresh royal jelly. Vacuum freeze-drying not only maximally preserves the quality of royal jelly, but also has significant advantages in terms of storage, transportation, usage and stability. However, the differences between lyophilized royal jelly and fresh royal jelly are still not clear.

## 5. Conclusions

In conclusion, our results showed that RJ can extend the lifespan of *C. elegans*, enhancing its growth rate and motility and boosting its reproductive capacity. The activity of RJ on *C. elegans* is related to its freshness, while RJ also contains stable active components that are independent of freshness. Next, it is necessary to study the dynamic variation in the composition and functions of RJ under different storage conditions. Moreover, we will explore effective methods for accurately assessing the freshness of RJ, thereby ensuring product quality, stability and reliability.

## Figures and Tables

**Table 1:** All primer sequences of *C. elegans* used in RT-qPCR experiments.

Gene	Gene ID	Forward (5′→3′)	Reverse (5′→3′)
*daf16*	172981	5′-CGCTTCTTCATCGGCTCTTC-3′	5′-CTTTCGAACAACACCAGGGG-3′
*sir2.1*	177924	5′-TTCAGAAGTTGCGGTCACAC-3′	5′-TGTGTTTGTTTCGGGTGCAT-3′
*skn1*	177343	5′-TCGCCTCTCTTCGGAATCTC-3′	5′-CTGGAAGCTCGTTGTCACTG-3′
*sod3*	181748	5′-GCGCTGAAATTCAATGGTGG-3′	5′-ATATCCCAACCATCCCCAGC-3′
*ctl2*	175085	5′-CTTCAACAAGGTCGGGAAGC-3′	5′-GTTCGGGAAGTGGATAGGGT-3′
*hsp16.2*	178659	5′-GGTGCAGTTGCTTCGAATCTT-3′	5′-TCTTCCTTGAACCGCTTCTTTC-3′
*hsf1*	173078	5′-TTTGCATTTTCTCGTCTCTGTC-3′	5′-TCTATTTCCAGCACACCTCGT-3′
*bec1*	177345	5′-ACGAGCTTCATTCGCTGGAA-3′	5′-TTCGTGATGTTGTACGCCGA -3′
*lgg1*	174050	5′-GCCGAAGGAGACAAGATCCG-3′	5′-GGTCCTGGTAGAGTTGTCCC -3′
*gpd1*	174603	5′-TCAAGGAGGAGCCAAGAAGG-3′	5′-CAGTGGTGCCAGACAGTTG-3′
*fat5*	180162	5′-TTGTCTGGAAGAATGTCGC-3′	5′-ATGAGCATCAGGAATACTCTCC-3′
*fat6*	178122	5′-AAAGATTGAGAAGGACGGC-3′	5′-CCGAACACATACAGAAGGAA-3′
*fat7*	179100	5′-CGAGCATTGCCAAGAAGAT-3′	5′-GCCGTTTGCCATTTAGCA-3′
*sbp1*	176574	5′-TCTCCACCACACCATACCT-3′	5′-GCCACTTGTTCAGGGTTCT-3′
*β-actin*	179534	5′-CGGAGCTGAACGGAAAACTC-3′	5′-TCAGTGTAGGCGAGGATTCC-3′

**Table 2:** Effect of RJ at different storage temperatures and dilution ratios on the lifespan of *C. elegans*.

Group		Number	Mean Lifespan (Days)	% of Control
−20 °C RJ	100 μg/mL	57	22.89 ± 0.59	116.37%
	50 μg/mL	54	21.54 ± 0.43	109.51%
	25 μg/mL	56	20.52 ± 0.34	104.32%
	12.5 μg/mL	54	19.84 ± 0.52	100.86%
4 °C RJ	100 μg/mL	53	20.12 ± 0.39	102.29%
	50 μg/mL	58	19.82 ± 0.28	100.76%
	25 μg/mL	56	19.45 ± 0.15	98.88%
	12.5 μg/mL	55	19.75 ± 0.46	100.41%
25 °C RJ	100 μg/mL	56	19.90 ± 0.22	101.67%
	50 μg/mL	52	19.51 ± 0.38	99.19%
	25 μg/mL	56	19.42 ± 0.63	98.73%
	12.5 μg/mL	53	19.73 ± 0.52	100.31%
PBS	Con.	55	19.67 ± 0.42	100%

## Data Availability

The original contributions presented in the study are included in the article; further inquiries can be directed to the corresponding author.
